# Infected hepatic cyst following pancreatoduodenectomy treated with transhepatic drainage and minocycline hydrochloride injection: A case report

**DOI:** 10.1016/j.ijscr.2020.02.008

**Published:** 2020-02-06

**Authors:** Yusuke Takahashi, Hitoshi Seki

**Affiliations:** Department of Digestive Surgery, Nagano Municipal Hospital, Nagano, 381-8551, Japan

**Keywords:** CT, computed tomography, AST, aspartate aminotransferase, ALT, alanine aminotransferase, ALP, alkaline phosphatase, γ-GTP, γ-glutamyltranspeptidase, CRP, C-reactive protein, WBC, white blood cell, SBT/ABPC, sulbactam/aminobenzyl penicillin, Pancreatoduodenectomy, Cysts, Minocycline, Infection, Drainage

## Abstract

**Introduction:**

Asymptomatic hepatic cysts, often observed on computed tomography, have never been reported as a risk factor for infection following pancreatoduodenectomy. Symptomatic liver cysts are treated surgically or non-surgically. We encountered a case of infected hepatic cyst following pancreatoduodenectomy.

**Presentation of case:**

An 88-year-old woman underwent pancreatoduodenectomy for adenocarcinoma in the duodenum papilla of Vater. She was discharged (postoperative day 23), following an uneventful course. However, 7 days later, she was re-admitted because of a high fever; postoperative cholangitis was suspected. A peripheral-venous blood bacterial culture was negative. Her C-reactive protein level and white blood cell count were 0.57 mg/dl and 5290/μl, respectively. Antibiotics were administered, but her high fever only temporarily decreased. Contrast-enhanced computed tomography showed ring enhancement surrounding the largest of several hepatic cysts. Percutaneous transhepatic drainage was performed; thereafter, minocycline hydrochloride was repeatedly injected into the infected cyst through the drain to prevent infection recurrence. The drain was removed 49 days after drainage. On contrast-enhanced computed tomography at 6 months postoperatively, the infected hepatic cyst had reduced in size from 6.0 to 1.7 cm and no findings of bile duct stenosis were observed. No further infectious events have been observed.

**Discussion:**

Relatively large hepatic cysts may be at risk of infection following pancreatoduodenectomy and, in particular, biliary reconstruction.

**Conclusion:**

Transhepatic drainage, rather than antibiotic administration, is essential, and minocycline hydrochloride injection into infected cyst that have no evidence of communication with the biliary tree is effective in preventing infection recurrence, without the complication of bile duct stenosis.

## Highlights

• Large hepatic cysts may be at risk of infection following pancreatoduodenectomy.
• Percutaneous transhepatic drainage, rather than antibiotic administration, is essential treatment.
• Minocycline hydrochloride injection into the infected cyst is also effective.

## 1 Introduction

Simple hepatic cysts are often observed on computed tomography (CT) or abdominal echography, with a frequency of 2.5%–18% in the general population [1,2]. Most hepatic cysts are asymptomatic; the frequency of symptomatic cysts, which present with hemorrhage, rupture, and infection, has been reported as only 5% [1]. Because of the rarity of symptomatic non-parasitic hepatic cysts, the risk factors of infected hepatic cysts have not been discussed. Therefore, asymptomatic hepatic cysts have never been reported as at risk of infection following pancreatoduodenectomy.

We encountered a case of infected hepatic cyst following pancreatoduodenectomy, which was treated with perihepatic drainage and minocycline hydrochloride administration. To the best our knowledge, this is the first reported case to suggest a risk of infected hepatic cyst following biliary reconstruction, such as that after pancreatoduodenectomy. This case report has been written in line with the SCARE criteria [3].

## 2 Presentation of case

**Fig. 1:**
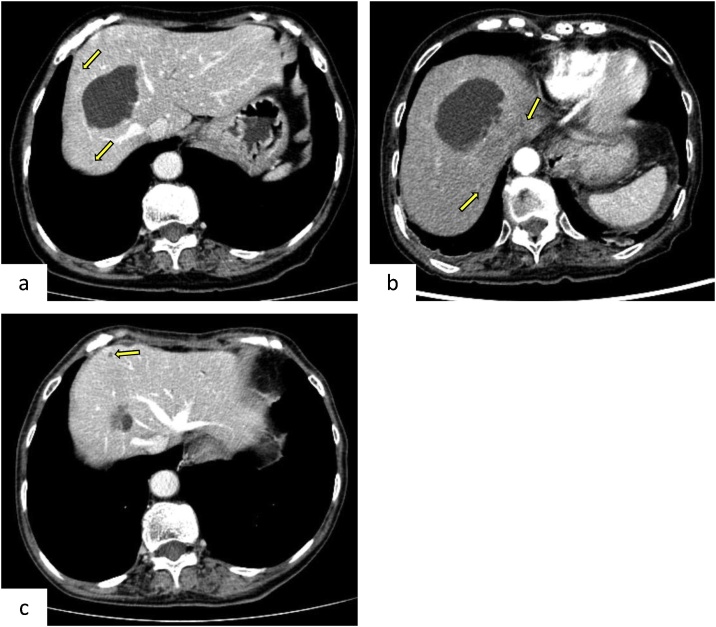
Contrast-enhanced abdominal computed tomography (CT). The arrow indicates the asymptomatic cyst. a. Preoperative CT shows a hepatic cyst, measuring 6.0 × 3.0 cm, in segment VIII. b. The border of the hepatic cyst is slightly enhanced on CT at 3 days after readmission. c. Repeated sclerotherapy using minocycline hydrochloride decreases the infected hepatic cyst from 6.0 to 1.7 cm.

An 88-year-old woman underwent pancreatoduodenectomy with modified Child reconstruction for an adenocarcinoma in the papilla of Vater of the duodenum, which was diagnosed by upper gastrointestinal endoscopy. Preoperative CT showed several asymptomatic hepatic cysts, the largest of which was 6.0 × 3.0 cm and located in segment VIII of the liver (Fig. 1a). She had no history of cholangitis, infected liver cysts, or obstructive jaundice preoperatively. Her postoperative course was uneventful, and she was discharged on postoperative day 23.

However, she was re-admitted to our hospital due to a high fever (39.6 °C), without other symptoms, on day 7 after discharge. Plain CT from the chest to the pelvis did not reveal the origin of the fever. Results of the biochemical examinations, including total bilirubin, aspartate aminotransferase (AST), alanine aminotransferase (ALT), alkaline phosphatase (ALP), γ-glutamyltranspeptidase (GTP), amylase, C-reactive protein (CRP), and white blood cell (WBC) counts, were all within normal ranges. A bacterial culture of peripheral-venous blood was negative. We suspected postoperative cholangitis, and antibiotics (sulbactam/aminobenzyl penicillin [SBT/ABPC] 1.5 g) were administered intravenously twice a day. Although her body temperature temporarily decreased, it subsequently increased to 39.1 °C, without any other symptoms.

**Fig. 2:**
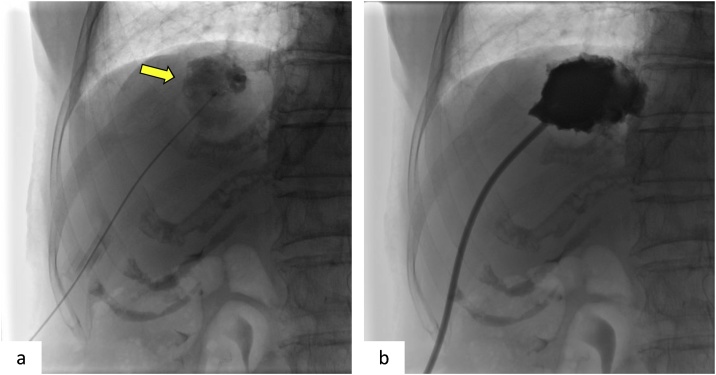
Percutaneous transhepatic drainage. a. Percutaneous transhepatic drainage is performed for the infected hepatic cyst (arrow) in segment VIII. b. An 8-French pigtail catheter is placed.

Contrast-enhanced abdominal CT at 3 days after readmission showed a slightly enhanced border around the largest hepatic cyst, in segment VIII of the liver; the hepatic cyst was also slightly enlarged on preoperative CT (Fig. 1b). As we suspected, the hepatic cyst was infected; percutaneous transhepatic drainage of the cyst was performed by the interventional radiologist, and an 8-French pigtail catheter was placed (Fig. 2a, b). About 15 ml of slightly turbid fluid was aspirated. Her high temperature immediately decreased without antibiotic administration. *Escherichia coli* was identified in a bacterial culture of the intracystic fluid.

**Fig. 3:**
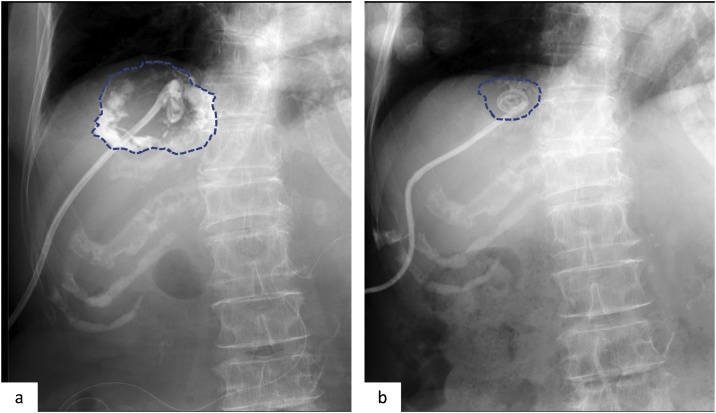
Sclerotherapy using minocycline hydrochloride under radiography. The dotted line indicates the target cyst. a. Initial sclerotherapy. Minocycline hydrochloride (200 mg) dissolved in 20 cc of saline is injected through the catheter. b. Before catheter removal. The cyst is greatly decreased in size compared to that after the initial sclerotherapy.

Starting at 7 days after drainage, minocycline hydrochloride (200 mg), dissolved in saline (5–20 cc) with 1% lidocaine (3 ml), was administered via the drain under radiography every other day to prevent infection recurrence (Fig. 3a). The drain was clamped after drug administration and re-opened 3 h later. The infected cyst gradually decreased in size; accordingly, the amount of minocycline administered was reduced (Fig. 3b). The minocycline hydrochloride treatment was repeated 8 times until she was discharged at 17 days after drainage; thereafter, it was performed once per week in an outpatient clinic. The cyst continued to gradually decrease in size, and the drain was removed at 49 days after drainage. During this period, she tolerated the treatment well. On contrast-enhanced abdominal CT at 6 months after surgery, the cyst measured 1.7 × 1.7 cm, and no evidence of primary cancer recurrence was observed (Fig. 1c). As of yet, no re-infection of the hepatic cyst or postoperative cholangitis has been observed.

## 3 Discussion

To the best of our knowledge, this is the first reported case of infected hepatic cyst following pancreatoduodenectomy in the English literature. However, rare cases of liver abscess following biliary enteric anastomosis have been reported [4]. As the patient did not have a history of infected hepatic cysts before the surgery, the pancreatoduodenectomy, and in particular, the biliary tract reconstruction, might be responsible for this rare entity.

Because the sphincter of Oddi, which provides a barrier against reflux, is resected, the occurrence rate of postoperative cholangitis after pancreatoduodenectomy is 6%–25% [[5], [6], [7]]. Abnormal liver function test results, namely, elevated serum AST, ALT, ALP, and γ-GTP levels, as well as fever and other evidence of an inflammatory response on a laboratory test, such as increased WBC count and CRP level, are useful for the diagnosis of acute cholangitis [8]. However, in the present case, only a high fever (39.6 °C) on readmission was observed; all laboratory data were within normal ranges. Infected liver cyst itself may not influence the liver function, consequently, leading to levels of AST, ALT, ALP, and γ-GTP that are within normal ranges. The reason for the within-normal-range WBC count and CRP level regardless of bacterial infection is unclear. We administered SBT/ABPC (3 g/day) under the diagnosis of postoperative cholangitis, albeit with atypical clinical findings. *E. coli, Klebsiella spp*, and *Enterococcus spp* are responsible for acute cholangitis, and postoperative cholangitis following biliary reconstruction is usually improved by antibiotic administration [9]. However, her high fever was only temporarily decreased by antibiotic administration, increasing again up to 40 °C.

Abdominal echography and contrast-enhanced CT are useful in the diagnosis of infected hepatic cysts [10]. In the present study, contrast-enhanced CT, but not plain CT, revealed findings suggesting an infected hepatic cyst. Ring enhancement of a hepatic cyst is a typical finding of infected hepatic cysts, and in this respect, the present case is compatible with previously reported cases.

Considering the cultured *E. coli* from the cystic fluid and the fact that the patient never experienced infected cysts before surgery, we consider the pancreatoduodenectomy with modified Child reconstruction as responsible for this entity. A retrograde infection from biliary reconstruction (hepaticojejunostomy) was suspected. Although she had several hepatic cysts, only the largest cyst was infected. Thus, a relatively large hepatic cyst (e.g., more than 5 cm) may be at risk of infection following surgery including biliary reconstruction. Relatively large cysts have greater fluid-retention capacity than do small liver cysts, which may allow proliferation of bacteria derived from the small intestine following biliary reconstruction, leading to a clinically relevant infected cyst with high temperature.

Symptomatic hepatic cysts can be treated surgically or non-surgically to prevent recurrence [[10], [11], [12], [13], [14]]. Surgical interventions, such as fenestration and hepatectomy, may be more effective than non-surgical interventions in terms of the recurrence rate, but are more invasive. In the present case, the high temperature was immediately decreased by percutaneous transhepatic drainage, without antibiotics. As a non-surgical intervention, sclerotherapy using ethanol may be less invasive than sclerotherapy using minocycline hydrochloride, but there is a future risk of biliary stenosis. As retrograde infection from biliary reconstruction was suspected in the present case, we selected minocycline hydrochloride as sclerotherapy, even though extrahepatic biliary tracts were not depicted on cystography. Minocycline hydrochloride is an acid that causes a reaction in wall cells with fibrosis and wall adhesions, and irritates the peritoneum [15]. Therefore, local anesthesia is necessary. In this case, minocycline with 1% lidocaine was administered.

The optimal amount of minocycline and the administration interval differ among reported cases [9,13]. According to Danza et al. [14] the quantity of minocycline hydrochloride should equal 1 mg per ml of the volume of the infected cyst. Repeated sclerotherapy using minocycline hydrochloride gradually decreased the cyst size, and we adjusted the amount of minocycline hydrochloride according to the decreased cyst size. No complication or recurrence was observed during and after sclerotherapy.

## 4 Conclusions

Relatively large asymptomatic hepatic cysts may be at risk of infection following pancreatoduodenectomy. Abdominal echography or contrast-enhanced CT is quite important in the diagnosis of this entity. Transhepatic drainage and sclerotherapy using minocycline hydrochloride are effective treatment options in such cases.

## Sources of funding

This research did not receive any specific grant from funding agencies in the public, commercial, or not-for-profit sectors.

## Ethical approval

Our institutional review board waived the requirement of ethical approval for this case report.

## Consent

Written informed consent was obtained from the patients for publication of this case report and accompanying figures. A copy of the written consent is available for review by the Editor-in-Chief of this journal on request.

## Author contribution

YT contributed to the study concept, design, and writing of the case report. HS participated in the treatment of the patient and drafted the manuscript. HS critically revised the manuscript. All authors read and approved the final manuscript.

## Registration of research studies

This case report was not registered in a publicly accessible database.

## Guarantor

Yusuke Takahashi accepts full responsibility for the work and the conduct of the case report, had access to the data, and controlled the decision to publish.

## Provenance and peer review

Not commissioned, externally peer-reviewed

## Declaration of Competing Interest

There are no conflicts of interest.
